# The effects of an anti-inflammatory diet alone or in combination with acupuncture on mental health, anthropometric indices, and metabolic status in diabetic patients with depression: a randomized, controlled clinical trial

**DOI:** 10.1038/s41387-025-00373-y

**Published:** 2025-05-02

**Authors:** Pardis Irandoost, Amir Firouzjaei, Javad Heshmati, Erfan Sadeghi, Mohammad Hossein Ayati, Nazli Namazi

**Affiliations:** 1 Department of Nutrition, School of Public Health, Iran University of Medical Sciences, Tehran, Iran; 2 Wellth by Medcare, Medcare Hospitals & Medical centres, Dubai, United Arab Emirates; 3 University of Ottawa, Heart Institute, Ottawa, ON Canada; 4 Department of Biostatistics, School of Medicine, Shiraz University of Medical Sciences, Shiraz, Iran; 5 Department of Traditional Medicine, School of Persian Medicine, Tehran University of Medical Sciences, Tehran, Iran; 6 Research Center for Integrative Medicine in Aging, Aging Research Institute, Tabriz University of Medical Sciences, Tabriz, Iran; 7 Beijing University of Chinese Medicine (BUCM), Beijing, China; 8 Diabetes Research Center, Endocrinology and Metabolism Clinical Sciences Institute, Tehran University of Medical Sciences, Tehran, Iran

**Keywords:** Medical research, Endocrinology

## Abstract

**Background:**

The present clinical trial examined the efficacy of an anti-inflammatory diet combined with acupuncture compared to an anti-inflammatory diet alone and standard treatment in depressed patients with type 2 diabetes (T2DM).

**Methods:**

In this 8-week randomized controlled clinical trial, 90 patients with T2DM who were experiencing mild to moderate depression were included. The participants were randomly assigned to one of three groups: (i) acupuncture combined with an anti-inflammatory diet, (ii) an anti-inflammatory diet alone, and (iii) standard treatment. The combination therapy group received acupuncture therapy twice a week. Mental health outcomes, biochemical parameters, dietary intake, and anthropometric indices were assessed at baseline and the end of the trial.

**Results:**

Of the 90 diabetic patients, 83 completed the intervention. Acupuncture therapy combined with diet resulted in an ~20% reduction in depression and anxiety, 4.28 and 0.82% reduction in waist circumference (WC) and HbA1C levels, respectively at the end of the trial. This combination therapy also significantly decreased WC (*p* = 0.04) and HbA1c levels (*p* = 0.008), while increasing high-density lipoprotein cholesterol concentrations (*p* = 0.02) compared to diet alone.

**Conclusion:**

Our findings indicate that acupuncture, in conjunction with an anti-inflammatory diet, may be more effective in enhancing mental health, reducing HbA1C levels, and decreasing abdominal obesity compared to an anti-inflammatory diet alone in patients with T2DM experiencing mild-to-moderate depression after 8 weeks. However, further clinical trials with larger sample sizes and extended durations are recommended to confirm the efficacy of this adjunctive therapy.

## Introduction

Diabetes mellitus, the most prevalent and clinically significant metabolic disorder, represents a global pandemic and poses a major healthcare challenge worldwide. Based on the latest report from the International Diabetes Federation (IDF), 537 million adults are currently affected by diabetes [1]. Behavioral [2] and psychological interventions [3] have been shown to play crucial roles in managing glucose levels and preventing complications associated with diabetes [4]. A strong link between diabetes and depression highlights the importance of interventions that positively affect both conditions [5]. Given the high prevalence of polypharmacy among patients with diabetes [6], non-pharmacological treatments, such as dietary modifications and alternative therapies, are expected to be more effective than pharmacological approaches [7]. Factors such as the cost of treatment, side effects of medications—particularly antidepressants—and the negative psychological impacts of polypharmacy on patients and their families further highlight the necessity of exploring non-pharmacological therapies [8].

Inflammation is a common factor that contributes to both depression and complications associated with diabetes [9]. Diet and lifestyle are modifiable factors that can help reduce inflammatory markers [10]. Previous studies have demonstrated the positive effects of healthy diets and acupuncture on metabolic health [10–16] and psychological well-being [17–19]. Evidence suggests that acupuncture may influence inflammation and depression through the release of neurotransmitters, modulation of the immune response, reduction of stress hormones, and enhancement of blood circulation [20].

A variety of nutrients, including vitamins A, E, B6, B9, B12, C, and D, as well as beta-carotene, anthocyanidins, selenium, zinc, and magnesium, contribute to anti-inflammatory properties. Additionally, certain spices such as rosemary, ginger, saffron, and turmeric, along with dietary components like fiber, omega-3 fatty acids, and monounsaturated fatty acids, and food items such as garlic and onion, are also recognized for their anti-inflammatory effects [21].

It is expected that adherence to an anti-inflammatory diet can improve mental health, enhance metabolic status, and increase patient compliance compared to pharmacological treatments [22]. However, to the best of our knowledge, there have been no clinical trials that have specifically examined the effects of an individualized anti-inflammatory diet on patients with depression and type 2 diabetes (T2DM). Furthermore, there is a scarcity of clinical trials that have compared the efficacy of an anti-inflammatory diet with acupuncture therapy. Additionally, there is a limited number of clinical trials that have primarily focused on reducing pro-inflammatory dietary components to manage depression. Given the insufficient evidence as well as the conflicting findings related to each type of intervention, further clinical trials are necessary to yield more conclusive results.

Accordingly, the current clinical trial aimed to compare the efficacy of an anti-inflammatory diet combined with acupuncture to two other treatment modalities: an anti-inflammatory diet alone and a standard treatment. Our study also focused on evaluating the effects of these interventions on mental health, metabolic status, and anthropometric indices in depressed patients with T2DM.

## Methods

In this present randomized controlled clinical trial, we examined the effects of an anti-inflammatory diet, both alone and in combination with acupuncture, on mental health, anthropometric indices, and metabolic status in diabetic patients with depression.

### Eligibility criteria

The inclusion criteria were as follows: (i) both genders, (ii) aged 20–75 years, (iii) diagnosed with T2DM for more than six months, (iv) currently taking oral anti-diabetic medications, and (v) experiencing mild-to-moderate depression. Patients were excluded if they were using insulin or liraglutide, taking medicinal herbs or herbal remedies, antibiotics, antioxidant supplements (including multivitamins and minerals, vitamin E, vitamin C, and zinc), suffering from severe depression, having cardiovascular diseases, chronic liver or kidney disease, untreated thyroid disorders, or any other diabetes-related complications that could lead to inflammatory and oxidative conditions. Additionally, individuals with other types of diabetes or those taking psychiatric medications were excluded. Pregnancy and lactation were also considered exclusion criteria.

### Intervention

Eligible patients were randomly selected from those referred to a diabetes clinic affiliated with Tehran University of Medical Sciences. Before the trial commenced, the study was explained to the eligible individuals, and if they volunteered to participate, they signed a written informed consent form.

Eligible individuals were assigned to one of three groups: (i) an individualized anti-inflammatory diet combined with acupuncture therapy, (ii) an individualized anti-inflammatory diet alone, or (iii) a control group receiving standard treatment and dietary recommendations. Importantly, all three groups received standard treatment, which included medications, a weight-loss diet for overweight and obese participants, and general healthy dietary recommendations, throughout the duration of the study. Patients were instructed to report any changes in the type and dosage of their medications during the study period.

### Diet

For each participant, an individualized diet tailored to their individual characteristics was designed by a nutritionist. The Mifflin equation [23] utilized to calculate the resting metabolic rate. Of the total calculated caloric intake, 50% was allocated to carbohydrates, 20% to protein, and 30% to fat. For participants who were overweight or obese, 500 kcal per day was deducted from the total calculated caloric intake.

To reduce the inflammatory potential of an individual’s overall diet, 45 food items [21] exhibiting pro-inflammatory and anti-inflammatory characteristics were considered for the calculation of dietary inflammatory factors (DII). To decrease dietary inflammation, foods and components with a lower DII were prioritized, while the intake of pro-inflammatory dietary components was restricted.

In general, considering the pro-inflammatory and anti-inflammatory food parameters, food sources, and the characteristics of the participants, an individualized anti-inflammatory diet was developed for both intervention groups.

### Acupuncture

Acupuncture was performed for two sessions (30 min in each session) per week for a period of 8 weeks. Acupoints were selected based on the WHO standard acupuncture point locations in the Western Pacific regions, and needle manipulation techniques were based on an acupuncture textbook in China. The main acupuncture points (*n* = 22) were as follows: DU20, LV3 (Bilateral), SP6 (Bilateral), ST36 (Bilateral), ST25 (Bilateral), SP15 (Bilateral), PC6 (Bilateral), HT7 (Bilateral), Sishencong (EX-HN 1), Yintang (EX-HN 3), Ren4, Ren6.

Our designed protocol for “Diabetic patients with Depression” emphasized addressing various dimensions, etiologies, and symptoms of depression while avoiding factors that have been shown to increase blood sugar levels.

### Sample size

In the present clinical trial with three study groups, type 2 diabetic patients with depression (mild to moderate) were examined, and our primary outcome was depression. The sample size was calculated using the G*Power analysis program. The true difference between the means was assumed to be 5 scores in depression. Considering ANOVA as a statistical test, the number of groups = 3, alpha error 0.05, power 80%, and effect size *f* = 0.33, 30 subjects are needed for each study group.

### Outcomes

In the present study, depression was our primary outcome, and biochemical parameters (FBS, HbA1c, TC, TG, LDL-C, HDL-C, high-sensitivity C-reactive protein (hs-CRP)), anxiety, and anthropometric indices (body weight, body mass index, WC), and dietary intake were our secondary outcomes.

### Recruitment

At the beginning of the study, eligible individuals were examined using the appropriate methods. Montgomery–Åsberg Depression Rating Scale (MADRS) questionnaire was administered through face-to-face interviews. Based on the scores obtained, individuals identified as experiencing mild-to-moderate depression (with scores ranging from 7 to 34) were included, while those with severe depression were referred to a psychologist. Importantly, all patients received standard treatment, and there were no changes in the dosage or type of medications throughout the study. Additionally, participants were instructed not to alter their physical activity levels during the study period. Since patients with mild-to-moderate depression typically do not receive antidepressant medications, we included only these individuals in our study.

Eligible patients were selected randomly from those referred to Diabetes clinics affiliated with Tehran University of Medical Sciences. Before starting the trial, the study was explained to eligible individuals, and if they wanted to participate, they would sign the written informed consent. Randomization: before the intervention, all eligible subjects participated in a two-week run-in period. Following this period, volunteers were randomly assigned (1:1:1) to one of three groups: (i) receiving an individualized anti-inflammatory diet combined with acupuncture therapy (*n* = 30), (ii) receiving only an individualized anti-inflammatory diet, and (iii) receiving a standard diet with dietary recommendations (control group) (*n* = 30).

Notably, volunteers were randomized and allocated to each group based on the severity of depression (mild, moderate). The randomization was carried out using an online system (https://www.sealedenvelope.com/). Once the randomization has been made, each patient is given a code with which she/he was identified throughout the study. The randomization numbers were assigned consecutively. Randomization was not exposed to those conducting the study and was provided in sealed opaque envelopes with successive numbers. The envelope was opened after signing the informed consent form, and the patients complied with the eligibility criteria.

### Blinding

Due to the nature of intervention in this study, complete blinding is not feasible. Only statistical analysis was conducted in blinded manner.

### Data collection

Participants were followed for 8 weeks to examine any changes in mental health (depression, anxiety), metabolic parameters (FBS, HbA1c, TC, TG, LDL-C, HDL-C, hs-CRP), anthropometric indices (body weight, body mass index (BMI), waist circumference (WC) and body composition) and dietary intake following the intervention. At the beginning and the end of the first and second months of the study, three 24-hour dietary recalls (2 work days, 1 weekend), a physical activity questionnaire, and anthropometric measurements with standard methods were obtained. In addition, at the beginning and the end of the study, depression and biochemical parameters were assessed. Visit descriptions are provided in Table 1.

**Table 1:** Visit descriptions at the beginning and throughout the study.

Time	Assessments
Visit 0	Screening for depression
Visit 1 (baseline)	The assessments of (i) anthropometric indices, (ii) dietary intake, (iii) physical activity, (iv) biochemical assessment, and (vi) anxiety.
Visit 2 (at the end of the fourth week)	The assessment of (i) anthropometric indices, and (ii) dietary intake.
Visit 3 (at the end of the study)	The assessment of (i) anthropometric indices, (ii) physical activity, (iv) dietary intake, (v) biochemical assessment, and (vi) mental assessment.

### The assessments of mental health

Depression and anxiety will be examined using MADRS and the Beck questionnaire, respectively. Physical activity information was obtained through the International Physical Activity Questionnaire [24] at baseline and at the end of the study via a face-to-face interview. To examine depression, the MADRS questionnaire was used. In the present study, we applied the Persian form of MADRS; Notably, its validity and reliability were evaluated earlier [25]. The MADRS questionnaire assesses the following ten items: (1) apparent sadness, (2) reported sadness, (3) inner tension, (4) reduced sleep, (5) decreased appetite, (6) concentration difficulties, (7) lassitude, (8) inability to feel, (9) pessimistic thoughts, and (10) suicidal thoughts. Each of these ten items is rated by a clinician on a seven-point Likert scale, and the scores are summed to produce a total score ranging from 0 to 60. A higher score indicates a greater severity of depression. Usual cutoff points are as follows: 0 to 6: normal, 7 to 19: mild depression, 20 to 34: moderate depression, >34: severe depression. Accordingly, only patients with a score between 7 and 34 were included.

To assess anxiety, the Beck questionnaire (21 items) was used. Individuals with a score of 0–7 were considered to have the least anxiety, and those with a score of 26–63 were considered to have severe anxiety.

### Anthropometric indices measurements

A digital weighing scale (Seca 725 GmbH & Co., Hamburg, Germany) was used for body weight measurement to the nearest 0.1 kg. Body weight and height (precision measurement: 0.5 cm) were measured using standard methods. BMI was computed by dividing the weight (kg) by the square of height 11 (m). WC was measured to the nearest 0.5 cm at the midpoint between the lower border of the rib cage and the iliac crest using a flexible tape (cm).

### Biochemical measurement

At the beginning and the end of the study, after 12–14 h of fasting, 10 cc of blood was collected from the left arm arterial of study participants. Blood was taken in a sitting posture. The serum was separated by centrifugation and stored in the freezer at −70 °C until the measurement of the biochemical indicators. Notably, FBS and serum lipid levels were immediately measured after blood sampling. All biochemical variables were examined by commercial biochemical kits according to the manufacturer’s protocol.

### Ethical and confidential issues

All participants provided written informed consent at the beginning of the study. All participant information is kept in a password-protected file with restricted access. To maintain participant confidence, a code identification number is used to identify data collection and forms. This research was conducted following the principles of the Declaration of Helsinki and was registered in the National Institute for Medical Research Development (NIMAD) with the Ethical Code of IR.NIMAD.REC.1398.340. The protocol was registered in the Iranian Registry of Clinical Trials (IRCTID: IRCT20110314006065N3).

### Statistical methods

Descriptive information was presented by mean ± Standard deviation (SD) or number (Percent), and was compared using Mann–Whitney *U* and Pearson Chi-Square tests between the study groups. Shapiro–Wilk test was also applied to assess the normality assumption within the groups. Furthermore, the Generalized Estimating Equations model adjusted for age, duration of diabetes, and total energy was performed to evaluate the between-group and within-group differences in terms of fat and anthropometric indices. In addition, generalized linear models adjusted for age, weight, duration of diabetes, total energy, and the outcome baseline value were used to compare the groups in terms of depression, anxiety, biomarkers, and nutritional parameters. Both models were followed by the least significant difference post-hoc test for pairwise comparisons between or within groups. All analyses were performed through IBM SPSS version 24, and *P* values <0.05 were considered statistically significant.

## Results

**Fig. 1:**
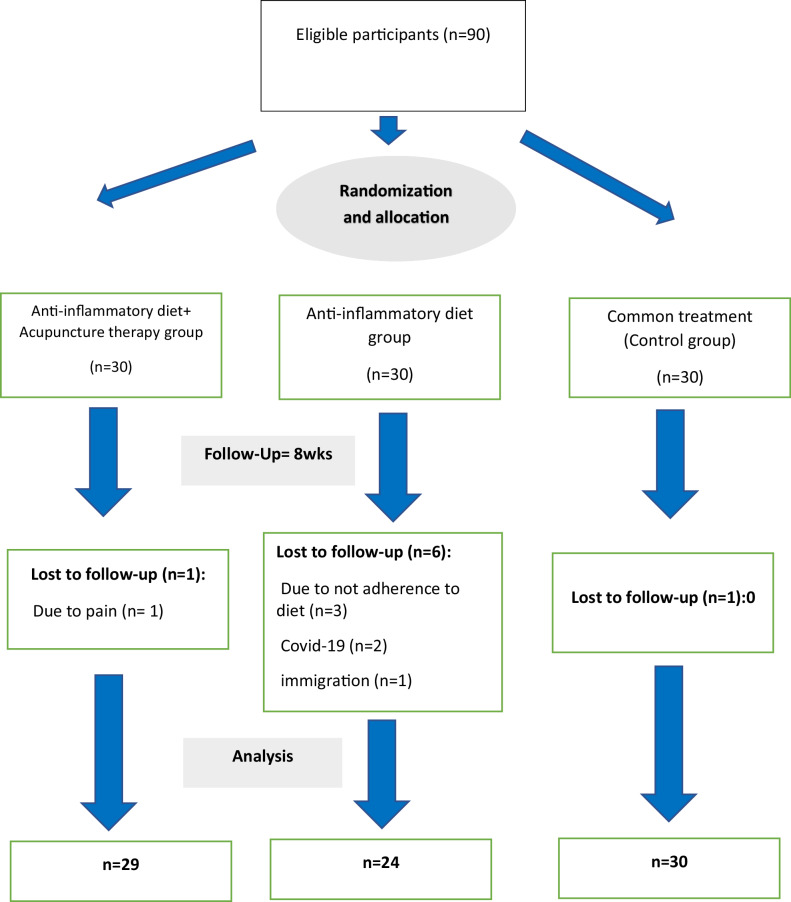
The study flow of the study.

Of 90 participants enrolled for the trial, 83 patients completed the trial. The study flowchart including the reasons for drop-out in each study group is presented in Fig. 1. The compliance rate to refer for acupuncture sessions was 95% and no severe adverse effects were reported. Only 1 patient in the acupuncture group did not complete the study due to pain.

The baseline characteristics of patients in the three study groups are comparable in Table 2. There were no significant differences in general characteristics except in disease history (*p* < 0.001) and having allergies (*p* = 0.08) among groups. Both genders, 55% women and 45% men, participated in this study.

**Table 2:** Demographic information of the participants.

		Acupuncture +diet *n* = 30	Diet alone *n* = 30	Standard treatment (control) *n* = 30	*P* value
Age (year)		47.7 ± 3.08	48.23 ± 3.9	47.77 ± 2.9	0.810^a^
Diabetes history (year)		10.29 ± 7.91	9.8 ± 7.79	8.67 ± 6.02	0.859^a^
Sleep duration (hour)		7.1 ± 1.42	7.17 ± 1.42	7.7 ± 1.21	0.879^a^
Education	Illiterate	0 (0)	1 (3.3)	0 (0)	0.712^b^
	Under diploma	10 (33.3)	10 (33.3)	7 (23.3)	
	Diploma	14 (46.7)	11 (36.7)	14 (46.7)	
	MSc and higher	6 (20)	8 (26.7)	9 (30)	
Marital status	Single	1 (3.3)	2 (6.7)	2 (6.7)	0.352^b^
	Married	27 (90)	28 (93.3)	28 (93.3)	
	Widow/divorced	2 (6.7)	0 (0)	0 (0)	
Family history of diabetes	Yes	24 (80)	20 (66.7)	16 (53.3)	0.091^b^
	No	6 (20)	10 (33.3)	14 (46.7)	
Other disease history	yes	22 (73.3)	20 (66.7)	5 (17.2)	<0.001^b^
	no	8 (26.7)	10 (33.3)	24 (82.8)	
	no	18 (60)	18 (60)	25 (86.2)	
Allergy	yes	6 (20)	1 (3.3)	0 (0)	0.008^b^
	no	24 (80)	29 (96.7)	30 (100)	

^a^Mann–Whitney *U* test, ^b^Pearson Chi-Square test.

The comparison of anthropometric indices (Table 3) revealed no significant differences among study groups at baseline. Regarding BMI, there were significant differences in BMI between the group receiving only an anti-inflammatory diet and the control group (*p* = 0.01). After adjusting for baseline BMI, no significant differences were found at the end of the trial. As shown in Table 3, at the beginning of the study, WC was different among the study groups. However, after adjusting WC in the middle and at the end of the trial, significant differences remained between the intervention groups and the control group (*p* < 0.05 in both). In addition, at the end of the study, an anti-inflammatory diet with (*p* = 0.04) and without acupuncture (*p* = 0.003) reduced WC compared to the standard treatment. A comparison of the two intervention groups also showed that an anti-inflammatory diet, along with acupuncture, compared to diet alone, led to more reduction (4.6 vs.1.5%) in abdominal obesity at the end of the trial.

**Table 3:** Comparison of anthropometric indices between the treatment groups and within the follow-up period.

Variable	Time	Acp + Diet (^a^)	Diet alone (^b^)	Control (^c^)	*P* Within^a^	*P* Within^b^	*P* Within^c^	*P* Between at Baseline	*P* Between at Middle	*P* Between at End
Fat mass (%)	Baseline	41.92 ± 8.13	42.88 ± 7	43.09 ± 3.24	*P* 12 = 0.587	*P* 12 = 0.583	*P* 12 = 0.177	*P*^ab^ = 0.593	*P*^ab^ = 0.523	*P*^ab^ = 0.059
	Middle	42.31 ± 6.88	43.6 ± 6.04	43.49 ± 3.1	*P* 13 = 0.003	*P* 13 = 0.306	*P* 13 = 0.101	*P*^ac^ = 0.857	*P*^ac^ = 0.839	*P*^ac^ = 0.801
	End	40.8 ± 8.16	44.01 ± 4.36	42.31 ± 3.79	*P* 23 = 0.067	*P* 23 = 0.482	*P* 23 = 0.004	*P*^bc^ = 0.667	*P*^bc^ = 0.635	*P*^bc^ = 0.068
Muscle mass (kg)	Baseline	25.62 ± 4.02	25.06 ± 3.47	24.57 ± 1.7	*P* 12 = 0.350	*P* 12 = 0.510	*P* 12 = 0.965	*P*^ab^ = 0.554	*P*^ab^ = 0.607	*P*^ab^ = 0.065
	Middle	25.21 ± 3.48	24.68 ± 2.93	24.58 ± 1.44	*P* 13 = 0.181	*P* 13 = 0.137	*P* 13 = 0.070	*P*^ac^ = 0.361	*P*^ac^ = 0.645	*P*^ac^ = 0.539
	End	25.89 ± 4.05	24.25 ± 2.39	25.02 ± 1.71	*P* 23 = 0.117	*P* 23 = 0.307	*P* 23 = 0.052	*P*^bc^ = 0.741	*P*^bc^ = 0.931	*P*^bc^ = 0.147
Visceral fat	Baseline	11.07 ± 4.7	9.57 ± 2.93	8.38 ± 1.86	*P* 12 = 0.013	*P* 12 = 0.020	*P* 12 = 0.555	*P*^ab^ = 0.123	*P*^ab^ = 0.260	*P*^ab^ = 0.101
	Middle	9.33 ± 3.12	8.58 ± 2.06	8.44 ± 1.98	*P* 13 = 0.011	*P* 13 = 0.014	*P* 13 = 0.949	*P*^ac^ = 0.001	*P*^ac^ = 0.095	*P*^ac^ = 0.032
	End	9.93 ± 3.93	8.58 ± 2.02	8.4 ± 2.02	*P* 23 = 0.280	*P* 23 = 0.788	*P* 23 = 0.795	*P*^bc^ = 0.040	*P*^bc^ = 0.603	*P*^bc^ = 0.518
Weight (Kg)	Baseline	80.91 ± 13.86	79.01 ± 11.91	77.09 ± 10.05	*P* 12 = 0.005	*P* 12 = 0.799	*P* 12 = 0.749	*P*^ab^ = 0.502	*P*^ab^ = 0.670	*P*^ab^ = 0.643
	Middle	77.17 ± 12.89	78.51 ± 10.97	77.04 ± 9.97	*P* 13 < 0.001	*P* 13 = 0.056	*P* 13 = 0.536	*P*^ac^ = 0.069	*P*^ac^ = 0.543	*P*^ac^ = 0.374
	End	77.8 ± 13.85	76.72 ± 11.05	77.01 ± 10.45	*P* 23 = 0.520	*P* 23 = 0.067	*P* 23 = 0.602	*P*^bc^ = 0.302	*P*^bc^ = 0.324	*P*^bc^ = 0.678
Waist circumference (cm)	Baseline	101.82 ± 7.51	106.72 ± 10.43	92.9 ± 11.61	*P* 12 < 0.001	*P* 12 = 0.285	*P* 12 = 0.724	*P*^ab^ = 0.042	*P*^ab^ = 0.001	*P*^ab^ = 0.003
	Middle	97.64 ± 8.23	105.69 ± 9.9	93.19 ± 10.78	*P* 13 < 0.001	*P* 13 = 0.113	*P* 13 = 0.913	*P*^ac^ < 0.001	*P*^ac^ = 0.055	*P*^ac^ = 0.049
	End	97.39 ± 8.31	105.12 ± 10.35	92.77 ± 10.95	*P* 23 = 0.863	*P* 23 = 0.180	*P* 23 = 0.233	*P*^bc^ < 0.001	*P*^bc^ < 0.001	*P*^bc^ < 0.001
Hip circumference (cm)	Baseline	112.22 ± 8.33	110.52 ± 8.95	108.47 ± 6.23	*P* 12 = 0.001	*P* 12 = 0.470	*P* 12 = 0.353	*P*^ab^ = 0.377	*P*^ab^ = 0.797	*P*^ab^ = 0.886
	Middle	109.4 ± 7.44	109.81 ± 8.51	108.71 ± 6.31	*P* 13 < 0.001	*P* 13 = 0.759	*P* 13 = 0.793	*P*^ac^ = 0.036	*P*^ac^ = 0.591	*P*^ac^ = 0.325
	End	110.04 ± 8.56	110.38 ± 8.88	108.43 ± 6.16	*P* 23 = 0.335	*P* 23 = 0.268	*P* 23 = 0.204	*P*^bc^ = 0.348	*P*^bc^ = 0.490	*P*^bc^ = 0.345
BMI (kg/m^2^)	Baseline	31.79 ± 4.73	32.23 ± 3.65	30.76 ± 2.7	*P* 12 = 0.028	*P* 12 = 0.699	*P* 12 = 0.758	*P*^ab^ = 0.715	*P*^ab^ = 0.217	*P*^ab^ = 0.332
	Middle	30.78 ± 4.19	32.05 ± 3.31	30.74 ± 2.68	*P* 13 < 0.001	*P* 13 = 0.111	*P* 13 = 0.190	*P*^ac^ = 0.055	*P*^ac^ = 0.333	*P*^ac^ = 0.351
	End	30.59 ± 4.78	31.69 ± 3.34	30.63 ± 2.86	*P* 23 = 0.717	*P* 23 = 0.220	*P* 23 = 0.220	*P*^bc^ = 0.017	*P*^bc^ = 0.022	*P*^bc^ = 0.049

Adjusted for age, duration of diabetes, and total energy.

*P* 12: Within-group comparison of baseline and middle, *P* 13: Within-group comparison of baseline and end, *P* 23: Within-group comparison of middle and end.

*P*^ab^: Between-group comparison of ^a^ and ^b^, *P*^ac^: Between-group comparison of ^a^ and ^c^, *P*^bc^: Between-group comparison of ^b^ and ^c^.

**Fig. 2:**
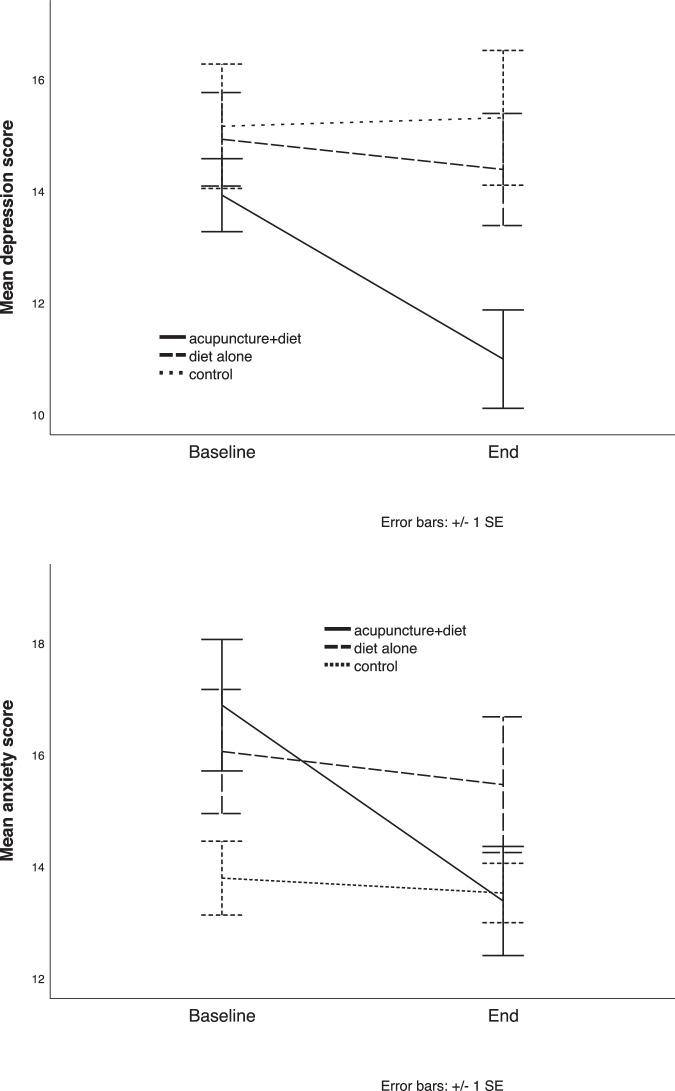
Percentage changes in depression and anxiety scores in the three study groups.

As it was represented in Table 4, acupuncture, along with an anti-inflammatory diet, improved depression and anxiety compared to diet alone and common treatment at the end of the intervention. Percentage changes in depression and anxiety scores in the three study groups are also shown in Fig. 2. Acupuncture plus diet therapy led to a 19.0% and 20.7% reduction in depression and anxiety scores at the end of the trial. Among biochemical parameters, the combination therapy only decreased HbA1C (*p* = 0.08) and increased HDL-C concentrations (*p* = 0.02) compared to diet alone. However, no significant differences in HDL-C concentrations were obtained between the combination therapy and common treatment (*p* = 0.08). The comparison with common treatment also showed the modulation of HbA1C (*p* = 0.03) with no significant changes in other parameters (Table 4).

**Table 4:** Comparison of depression, anxiety, and biochemistry markers between the treatment groups.

Variable	Time	Acupuncture + Diet (^a^)	Diet alone (^b^)	Control (^c^)	*P*^ab^	*P*^ac^	*P*^bc^
Depression	Baseline	13.9 ± 3.58	14.9 ± 4.58	15.13 ± 6.1	0.007	0.002	0.519
	End	10.97 ± 4.74	14.36 ± 5.02	15.29 ± 6.38			
Anxiety	Baseline	16.86 ± 6.33	16.03 ± 6.09	13.77 ± 3.62	<0.001	<0.001	0.687
	End	13.36 ± 5.17	15.44 ± 6.08	13.5 ± 2.81			
FBS (mg/dL)	Baseline	149.9 ± 36.45	154.23 ± 39.75	130.47 ± 24.15	0.258	0.974	0.296
	End	143.07 ± 24.51	152.61 ± 30.39	131.53 ± 25.85			
HbA1c (%)	Baseline	8.47 ± 1.29	8.6 ± 1.52	7.58 ± 0.91	0.008	0.030	0.813
	End	7.65 ± 0.99	8.3 ± 1.35	7.36 ± 0.86			
TC (mg/dL)	Baseline	132.57 ± 41.71	130.25 ± 38.17	123.35 ± 47.92	0.643	0.789	0.488
	End	134.79 ± 33.52	139.22 ± 26.01	129.3 ± 32.8			
TG (mg/dL)	Baseline	147.23 ± 57.35	165.93 ± 98.24	159.03 ± 101.47	0.481	0.248	0.071
	End	140 ± 56.88	163.35 ± 84.49	137.8 ± 81.82			
HDL (mg/dL)	Baseline	38.13 ± 5.46	42.07 ± 7.46	39.53 ± 10.65	0.027	0.086	0.691
	End	38.46 ± 7.71	44.78 ± 6.31	40.4 ± 9.07			
LDL (mg/dL)	Baseline	72.17 ± 28.54	79.34 ± 31.2	65.73 ± 30.55	0.234	0.511	0.662
	End	73.41 ± 27.64	73.73 ± 27.03	64.77 ± 22.96			
hs-CRP (mg/L)	Baseline	2.46 ± 3.45	2.45 ± 2.27	1.44 ± 0.6	0.639	0.810	0.639
	End	1.83 ± 2.3	1.6 ± 1.05	1.41 ± 0.56			

Adjusted for age, weight, duration of diabetes, total energy, and the outcome baseline value.

*P*^ab^: Between-group comparison of ^a^ and ^b^, *P*^ac^: Between-group comparison of ^a^ and ^c^, *P*^bc^: Between-group comparison of ^b^ and ^c^.

At baseline, no significant differences were found among the study groups. As it was expected, significant changes in total energy, macronutrients, types of fatty acids, and dietary fiber were observed at the middle and the end of the trial following the adherence to a low-calorie diet with anti-inflammatory characteristics (Appendix 1).

## Discussion

Our findings indicated that acupuncture, when combined with an anti-inflammatory diet, is more effective in reducing depression and anxiety scores, HbA1c levels, and abdominal obesity compared to an anti-inflammatory diet alone after 8 weeks. Additionally, the combination therapy resulted in increased serum levels of HDL-C, although it was not more effective than standard treatment. No significant differences were observed between the two intervention groups regarding dietary intake at the end of the trial.

From a clinical perspective, the synergistic interaction between an anti-inflammatory diet and acupuncture appears to enhance the effects of the diet alone on mental health, HbA1c levels, and abdominal obesity in diabetic patients with depression. Therefore, health providers can implement this adjuvant therapy for patients with T2DM, particularly those who suffer from depression and anxiety, to improve both their physiological and mental well-being.

In addition, no serious side effects were reported during the trial. Only two patients experienced pain and bruising following the acupuncture therapy, which resolved after several days. However, the pain resulted in only one individual dropping out of the combination therapy. Therefore, it can be a safe and effective therapeutic option for clinicians to manage patients with T2DM.

In the present trial, combination therapy resulted in a 0.8% reduction in HbA1C compared to the baseline. However, changes in FBS levels were minimal. Our findings regarding the effects of acupuncture on HbA1C were in line with several clinical trials [15, 16, 26]. In an RCT by Kazemi et al., conducted on subjects with T2DM, it was indicated that in the intervention group, which received acupoint stimulation (15 acupoints), HbA1C decreased significantly compared to the sham group after 10 weeks (14 sessions) [16]. Zhiyuan and his colleagues also reported that transcutaneous electric nerve stimulation reduced HbA1c and modulated lipid levels compared to the sham electrical-stimulation group after 2 months (five times/week) [26]. In addition, Firouzjaei et al., compared the effects of metformin and acupuncture combined therapy with metformin. They found that combination therapy reduced body weight and BMI, improved glycemic status, and lipid profile after 3 weeks (3 sessions/week) [15]. However, Li and his colleagues revealed that acupuncture was not effective on glycemic status in patients with diabetic gastroparesis [27]. Notably, the duration (1 week) and frequency of sessions were too short compared to other trials. Based on a systematic review and meta-analysis of 21 studies, compared with sham acupuncture or no acupuncture therapy (common treatment), acupuncture as a complementary therapy can reduce FBS (MD: 1.21 mmol/l, 95% CI: 1.56 to 0.87), 2 h blood sugar (MD: 2.13 mmol/l, 95% CI: 2.79–1.46), and HA1C (MD: 1.12 %, 95% CI: 1.62–0.62) and modulate lipid profile. However, this systematic review and meta-analysis recommended further well-designed studies with larger sample sizes to confirm the efficacy [28].

As mentioned in the methods section, our primary outcome was depression. Consequently, the selection of acupoints was specifically aimed at alleviating depressive symptoms. However, in the previously discussed clinical trials involving diabetic patients, the primary objective appeared to be the management of glycemic status. As a result, acupoints recognized for their glucose-lowering effects were prioritized. This difference in focus is likely one of the primary reasons for the varying changes observed in HbA1c and other metabolic parameters following the intervention.

Additionally, the current study recommended individualized anti-inflammatory diets for both intervention groups. When comparing the results of the diet with the common treatment, we found relatively similar findings to previous studies [10, 11, 13, 29]. These findings further confirm the positive effects of such diets on anthropometric indices and metabolic parameters.

Possible mechanisms for the positive effects of acupuncture on HbA1c include the following: (I) reduction in body weight and abdominal obesity, (II) inhibition of pancreatic beta-cell apoptosis, (III) enhancement of beta-cell function, (IV) increased insulin sensitivity and C-peptide levels, (V) reduction in insulin resistance through the activation of AMP-activated protein kinase and sirtuin 1/peroxisome proliferator-activated receptor γ coactivator 1α signaling, (VI) upregulation of nitric oxide synthase activity, (VII) improvement in glucose metabolism, (VIII) modulation of hormone levels, including insulin, glucocorticoids, and epinephrine, and (IX) reduction in oxidative stress and inflammatory parameters [15, 28, 30]. Chinese medicine believes that acupuncture can modulate glycemic status through acupoints that stimulate and regulate the spleen and stomach, the generation of the source of Qi and blood, and eventually generate fluid and quench thirst [23].

Notably, one of the parameters that plays a key role in HbA1C control is mental health. In the present clinical trial, the combination therapy significantly improved both depression and anxiety scores by the end of the study. However, adherence to an anti-inflammatory diet alone showed no positive effects compared to standard treatment. Evidence suggests that improvements in mental health can enhance adherence to dietary and medical recommendations among diabetic patients [31].

Our clinical trial concluded that combination therapy can improve depression and anxiety by 20%. It seems that positive effects of different types of acupuncture on depression and anxiety have been reported by earlier clinical trials, systematic reviews, and meta-analyses. In a randomized controlled clinical trial by Wei et al., patients with moderate depression received either electroacupuncture and antidepressants or antidepressants, and positive effects of combination therapy were obtained after 2 months of the intervention (3 times/per week). Additionally, after the combination therapy, significant differences in urinary specific metabolites, including malonic acid (fatty acid biosynthesis), glutathione (glutamate metabolism), cysteine (glutamate metabolism), and tryptophan (tryptophan metabolism were observed [18]. A systematic review and meta-analysis concluded that acupuncture, either in isolation or as a complementary therapy and along with pharmacological therapy, can be safe and effective for depression management [32]. A meta-regression of randomized controlled trials showed a direct association between the number of acupuncture sessions and symptom improvement in depressed individuals. Based on this review study, 36 acupuncture sessions have the optimal clinical response (66% reduction) in depression severity [33]. The differences in the percentage reduction of depression scores between our trial and the earlier study may be attributed to variations in the frequency of sessions, the duration of the study, the number and location of acupoints, the type of acupuncture used, the techniques employed by the acupuncturist, baseline mental health status, disease background, and other personal and environmental factors.

Our findings on anxiety were in line with a systematic review and meta-analysis. It showed beneficial effects of acupuncture on anxiety [17]. Based on the evidence, acupuncture can be helpful in repairing morphological and ultrastructural damage to neurons in the hippocampus and frontal lobe, reducing functional and structural damage to glial cells in the hippocampus and frontal lobe, strengthening functional connectivity, and improving structural connectivity [34]. Bai et al., also reported that acupuncture can regulate the expression of key proteins in the CaMK signaling pathway, which is involved in depression. This indicates that acupuncture at Shenting (GV24) and Baihui (GV20) is likely to alleviate depressive symptoms and stress by affecting the CaMK signaling pathway [35].

Although the precise mechanisms by which acupuncture affects mental health are not yet fully understood, evidence suggests that acupuncture may increase the levels of neurotransmitters, including serotonin, dopamine, and endorphins. It can also influence the hypothalamic-pituitary-adrenal axis, leading to a reduction in cortisol levels, which is often elevated in individuals with mental disorders. Additionally, the anti-inflammatory effects of acupuncture contribute to a decrease in inflammatory cytokines, thereby indirectly enhancing mental well-being. Furthermore, acupuncture may improve blood circulation, facilitate nutrient delivery, and promote balance within the autonomic nervous system, all of the above are potential pathways through which it alleviates symptoms of depression and anxiety.

Previous systematic reviews [14, 23, 24, 36] and clinical trials [37–40] reported positive effects of acupuncture on anthropometric indices and body compositions compared to common treatment. However, the findings are conflicting [41, 42]. A systematic review and meta-analysis by Wang et al. revealed that acupuncture has beneficial effects in the management of obesity combined with T2DM including reduction in BMI, WC, and fat mass rate [23]. An overview of 38 systematic reviews also demonstrated that acupuncture therapies and auricular acupoint stimulation can reduce body weight and BMI [14]. Acupuncture can also stimulate the areas of the brain that control metabolic changes [24]. For instance, Von Deneen et al. reported that acupuncture can affect basal metabolic rate and satiety hormones [43]. In addition, it can simulate some neurophysiological pathways, such as dopaminergic signaling, alter adipocyte morphology, apoptosis in adipocytes, gene expression in adipose tissue, reduction in gastrointestinal digestion and absorption, and the development of temporary pores [24]. Accordingly, impacts on several hormonal and neural pathways not only affect anthropometric indices and body composition but also improve biochemical parameters such as glycemic indicators, lipid profile, and inflammatory parameters. Reduction in insulin resistance induced by acupuncture can also be involved in weight reduction [24]. The mechanisms by which acupuncture therapy may improve anthropometric indices are not yet fully understood. It is likely that needle therapy affects biochemical markers associated with obesity, including obesity-related peptides (e.g., leptin and ghrelin), insulin resistance, and inflammatory markers. Additionally, acupuncture may suppress appetite by downregulating the expression of neuropeptide Y, slowing gastric emptying, and increasing serum levels of glucagon-like peptide-1. These factors could contribute to acupuncture’s potential to reduce anthropometric indices and enhance body composition. In the present clinical trial, acupoints were selected primarily to address depression. This focus may explain the lesser reduction in weight and BMI observed compared to previous trials targeting obesity. Furthermore, some patients undergoing combination therapy reported that adhering to the recommended diet was easier than their prior experiences with low-calorie diets. However, changes in appetite were not assessed through questionnaires or biochemical measurements.

Overall, personal characteristics, baseline levels of biochemical parameters, mental health status, adherence to medical and nutritional recommendations, study methodology, type of diet, acupoints, genetics, and race are key factors that can influence findings and should be taken into account when comparing with previous clinical trials. Evidence indicates that when baseline levels of variables are elevated, more significant changes can be observed following the intervention compared to situations where baseline values are only slightly above normal levels. Consequently, this may also lead to varying findings across different studies.

The present clinical trial has two main limitations that should be addressed. Inflammatory parameters, with the exception of hs-CRP, were not measured. Additionally, the sustainability of acupuncture’s effects following the completion of the study was not monitored. Nevertheless, this clinical trial appears to be the first to investigate the efficacy of an individualized anti-inflammatory diet in conjunction with acupuncture, as compared to diet alone and standard treatments, in diabetic patients with depression. Furthermore, both mental health (depression and anxiety) and physical health (biochemical parameters and anthropometric indices) of diabetic patients were assessed.

## Conclusion

Our findings indicate that acupuncture, when combined with an anti-inflammatory diet, is more effective in improving mental health, HbA1c levels, and abdominal obesity compared to an anti-inflammatory diet alone after eight weeks. Additionally, the combination therapy resulted in increased serum levels of HDL-C, although it was not more effective than standard treatment. The synergistic interaction between the anti-inflammatory diet and acupuncture appears to enhance the effects of the diet alone on mental health, HbA1c, and abdominal obesity in patients with T2DM who are experiencing mild to moderate depression. However, further clinical trials with larger sample sizes and extended durations are recommended to confirm the efficacy of this adjunctive therapy. Future studies should also examine acupuncture’s sustainability and potential long-term impact on mental and metabolic health.

## Supplementary information

Appendix 1- Comparison of nutritional (Energy residual adjusted) parameters between the treatment groups.

## Data Availability

The data from the present clinical trial are not publicly available due to patient privacy. However, it can be made available by a reasonable request sent to the corresponding author, Nazli Namazi, via email at nazli.namazi@yahoo.com
