# Supplementary material for: The effects of an anti-inflammatory diet alone or in combination with acupuncture on mental health, anthropometric indices, and metabolic status in diabetic patients with depression: a randomized, controlled clinical trial
Source: Nutr Diabetes. 2025 May 2;15:18. doi: 10.1038/s41387-025-00373-y (PMC12048675; doi:10.1038/s41387-025-00373-y)
Supplement: Supplementary file 1 — Appendix 1- Comparison of nutritional (Energy residual adjusted) parameters between the treatment groups. [file 41387_2025_373_MOESM1_ESM.docx]

**Appendix 1-** **Comparison of nutritional (Energy residual adjusted) parameters between the treatment groups.**

| **Outcome** | **Time** | **Acupuncture + Diet**  **(a)** | **Diet alone**  **(b)** | **Control**  **(c)** | **P ab** | **P ac** | **P bc** |
| --- | --- | --- | --- | --- | --- | --- | --- |
| Total energy (Kcal/day) | Baseline | 2396.34 ± 381.16 | 2413.31 ± 326.29 | 1957.75 ± 321.58 | 0.252 | <0.001 | <0.001 |
|  | End | 1425.07 ± 284.65 | 1513.47 ± 311.99 | 1907.94 ± 314.05 |  |  |  |
| Carbohydrate (g/day) | Baseline | 209.21 ± 44.23 | 201.33 ± 41.76 | 247.71 ± 56.81 | 0.225 | <0.001 | <0.001 |
|  | End | 307.14 ± 73.21 | 325.78 ± 56.33 | 253.56 ± 60.74 |  |  |  |
| Protein (g/day) | Baseline | 92.85 ± 30.23 | 89.05 ± 21.02 | 64.09 ± 16.4 | 0.061 | 0.004 | 0.209 |
|  | End | 46.12 ± 21.46 | 55.89 ± 21.56 | 62.91 ± 15.8 |  |  |  |
| Fat (g/day) | Baseline | 105.78 ± 39.34 | 95.83 ± 34.34 | 84.21 ± 25.04 | 0.806 | <0.001 | <0.001 |
|  | End | 47.62 ± 18.96 | 49.19 ± 18.24 | 74.81 ± 24.14 |  |  |  |
| Polyunsaturated fatty acids (g/day) | Baseline | 18.22 ± 10.2 | 19.58 ± 9.36 | 28.85 ± 13.99 | 0.848 | 0.022 | 0.010 |
|  | End | 19.69 ± 23.42 | 16.17 ± 6.66 | 26.5 ± 14.13 |  |  |  |
| Monounsaturated fatty acids (g/day) | Baseline | 14.66 ± 6.88 | 16 ± 7.51 | 24.72 ± 9.98 | 0.828 | <0.001 | <0.001 |
|  | End | 12.05 ± 7 | 12.08 ± 5.66 | 23.99 ± 9.86 |  |  |  |
| Saturated fatty acids (g/day) | Baseline | 15.5 ± 7.65 | 15.96 ± 7.03 | 18.99 ± 9.44 | 0.604 | <0.001 | <0.001 |
|  | End | 10.66 ± 5 | 11.38 ± 6.13 | 17.33 ± 5.8 |  |  |  |
| Dietary Fiber (g/day) | Baseline | 11.2 ± 4.97 | 14.04 ± 6.62 | 15.42 ± 5.07 | 0.643 | 0.001 | <0.001 |
|  | End | 9.94 ± 5.01 | 10.09 ± 3.24 | 14.86 ± 5.03 |  |  |  |

Adjusted for age, duration of diabetes, and the outcome baseline value.
